# Characteristics and Management of Patients with Refractory or Unexplained Chronic Cough in Outpatient Hospital Clinics in Spain: A Retrospective Multicenter Study

**DOI:** 10.1007/s00408-023-00620-y

**Published:** 2023-05-09

**Authors:** Ignacio Dávila, Luis Puente, Santiago Quirce, Ebymar Arismendi, Miguel Díaz-Palacios, Antonio Pereira-Vega, Alfredo de Diego, Juan Luis Rodriguez-Hermosa, Luis Cea-Calvo, Marta Sánchez-Jareño, Pilar López-Cotarelo, Christian Domingo

**Affiliations:** 1 Servicio de Alergia, Departamento de Ciencias Biomédicas Y del Diagnóstico, Facultad de Medicina, Hospital Universitario de Salamanca, Universidad de Salamanca, Salamanca, Spain; 2 Servicio de Neumología, Hospital Universitario Gregorio Marañón- Universidad Complutense, Madrid, Spain; 3 Servicio de Alergia, Hospital Universitario La Paz, IdiPAZ, Madrid, Spain; 4 Servei de Pneumologia, Hospital Clínic de Barcelona, Instituto de Salud Carlos III, CIBERES, IDIBAPS, Universitat de Barcelona, Barcelona, Spain; 5 Servicio de Alergia, Hospital Universitari I Politècnic La Fe, Valencia, Spain; 6 Servicio de Neumología, Hospital Juan Ramón Jiménez, Huelva, Spain; 7 Servicio de Neumología, Hospital Universitari I Politècnic La Fe, Valencia, Spain; 8 Servicio de Neumología, Departamento de Medicina, Hospital Clínico San Carlos, Universidad Complutense, Madrid, Spain; 9 Medical Affairs, MSD Spain, C. de Josefa Valcárcel, 38, 28027 Madrid, Spain; 10 Corporació Parc Taulí, Universitat Autònoma de Barcelona (UAB), Sabadell, Spain

**Keywords:** Refractory chronic cough, Unexplained chronic cough, Noninterventional study, Health resource utilization, Spain

## Abstract

**Purpose:**

Chronic cough (cough that persists for ≥ 8 weeks) can cause a range of physical symptoms and psychosocial effects that significantly impair patients’ quality of life. Refractory chronic cough (RCC) and unexplained chronic cough (UCC) are challenging to diagnose and manage, with substantial economic implications for healthcare systems.

**Methods:**

This retrospective multicenter non-interventional study aimed to characterize the profile and health resource consumption of patients with RCC or UCC who attended outpatient clinics at Spanish hospitals. Data were collected from medical records of patients with RCC or UCC for up to 3 years before study inclusion.

**Results:**

The patient cohort (n = 196) was representative of the chronic cough population (77.6% female, mean age 58.5 years). Two-thirds of patients (n = 126) had RCC. The most frequently visited doctors were pulmonologists (93.4% of patients) and primary care physicians (78.6%), with a mean of 5 visits per patient over three years’ observation. The most common diagnostic tests were chest x-ray (83.7%) and spirometry with bronchodilation (77.0%). The most commonly prescribed treatments were proton pump inhibitors (79.6%) and respiratory medications (87.8%). Antibiotics were prescribed empirically to 56 (28.6%) patients. Differences between RCC or UCC groups related mainly to approaches used to manage cough-associated conditions (gastroesophageal reflux disease, asthma) in patients with RCC.

**Conclusion:**

RCC and UCC are responsible for high health resource utilization in Spanish hospitals. Specific treatments targeting the pathological processes driving chronic cough may provide opportunities to reduce the associated burden for patients and healthcare systems.

**Supplementary Information:**

The online version contains supplementary material available at 10.1007/s00408-023-00620-y.

## Introduction

Chronic cough, defined as a cough that persists for longer than eight weeks, has an estimated 12-month period prevalence of around 5% according to recent population-based studies from the United States, Japan, Germany and Spain [1–4], and is one of the most common reasons for adults to seek medical attention [5, 6]. Chronic cough can cause physical symptoms (e.g., stress urinary incontinence, cough-related syncope, and dysphonia) and may lead to depression, social isolation, and difficulties in personal relationships, with a profoundly negative impact on an individual’s health-related quality of life [7–9].

Conditions frequently associated with chronic cough include postnasal drip (or upper airway cough syndrome), asthma, eosinophilic bronchitis, and gastroesophageal reflux disease (GERD). Other associated conditions are recent or active respiratory infection, smoking, and use of angiotensin-converting enzyme (ACE) inhibitors [5, 10, 11]. Troublesome coughing can also be triggered by low levels of innocuous stimuli such as changes in ambient temperature, taking a deep breath, laughing, talking on the phone and exposure to aerosols [12]. Although some individuals with chronic cough may benefit from trigger avoidance and/or usual treatment for cough-associated conditions, in a subset of patients either no underlying disease or etiology can be identified (unexplained chronic cough [UCC]), or cough persists despite thorough assessment and appropriate treatment of the underlying condition (refractory chronic cough [RCC]) [10].

The diagnosis and management of RCC and UCC can be a prolonged and challenging process for patients and doctors. Patients may visit a range of specialists, often repeatedly, in search of a specific diagnosis or treatment to alleviate the cough. Many patients undergo expensive or invasive medical procedures to reach a diagnosis, and diagnostic tests are often repeated during follow-up. Moreover, patients are often treated empirically with various drugs, with limited effectiveness and associated safety and tolerability issues that can lead to treatment discontinuation [9, 13, 14].

There are limited data specific to Spain regarding the consequences and burden of RCC and UCC on patients and the healthcare system. The 2020 population-based National Health and Wellness Survey conducted across 29 European countries found that Spanish respondents who self-reported chronic cough (579 of 7074 respondents in Spain) experienced inferior health status, poorer mental health, greater healthcare utilization, and lower productivity at work and home [4]. However, this study analyzed chronic cough patients in general and did not focus on the differences between RCC and UCC.

To obtain current data on the profile of patients with RCC and UCC, and associated health resource consumption (burden to the healthcare system), we undertook a study of patients with these conditions who had attended outpatient clinics at Spanish hospitals. An additional study objective was to explore the impact of RCC and UCC on various aspects of patients’ daily lives (burden to patients); this will be reported separately.

## Methods

### Study Design

This retrospective multicenter non-interventional study involved patients with RCC or UCC who attended outpatient clinics at representative hospitals from the National Healthcare System of Spain. Patients who were seen at the clinics between November 2020 and June 2022 were invited to participate in the study. The study protocol was reviewed and approved by the Research Ethic Committees of all participating hospitals. Enrolled patients provided signed informed consent before data collection began.

### Patients

Consecutive patients attending outpatient clinics were invited by their treating physician to enroll in the study if they were adults (> 18 years of age); had RCC or UCC according to physicians’ diagnosis; had been seen for chronic cough for the first time more than one year before study entry; had cough at the study visit date; and provided signed informed consent. Patients were excluded if they were current smokers or had stopped smoking less than one year before study entry; were receiving ACE inhibitors; had chronic cough related to chronic obstructive pulmonary disease, cancer, active infection, bronchiectasis, interstitial lung disease, cystic fibrosis, or Gilles de la Tourette syndrome; were participating in interventional studies or had conditions that, in the judgment of the treating physician, advised against participation (e.g., cognitive impairment, major depression, end-stage disease).

### Procedures

Patients’ clinical records were the primary source of information; no prospective data collection was performed. After providing consent, patients also completed a printed survey about the impact of chronic cough on their daily life activities and quality of life; these results will be reported separately. The predefined period for chart review was up to three years before study inclusion. Main items of interest were demographics, epidemiological variables, and comorbidities; cough characteristics; history and management of chronic cough including type of specialist(s) visited and number of visits, type and number of diagnostic tests performed, therapies used to treat chronic cough and its underlying condition, and antibiotic courses used empirically to treat chronic cough. The differentiation between RCC and UCC was based on the diagnosis reflected in the clinical chart, or according to physicians’ judgment after reviewing the clinical history, diagnostic tests performed and previous therapies used to treat chronic cough. No diagnostic test requirements or protocols were followed to categorize patients. There were no other specific procedures related to the study.

### Statistical Analysis

This was an exploratory study with no prespecified hypothesis. The sample size was calculated based on a conservative approach with 95% confidence, 7% precision, and an expected prevalence of 50% of any variable, yielding a sample size of 196 patients with RCC or UCC. No stratification (i.e., minimum number of each phenotype) was required between RCC and UCC.

For sample descriptions, quantitative variables are expressed as mean and standard deviation (SD) or median and interquartile range (IQR), and qualitative variables are expressed as frequency and percentage.

Results were compared between patients diagnosed with RCC or UCC. The Student t-test or analysis of variance test was used to compare continuous variables, and the chi-squared test or Fisher exact test was used to compare proportions.

All analyses were performed using the IBM SPSS 20.0.0 statistical program.

## Results

**Fig. 1:**
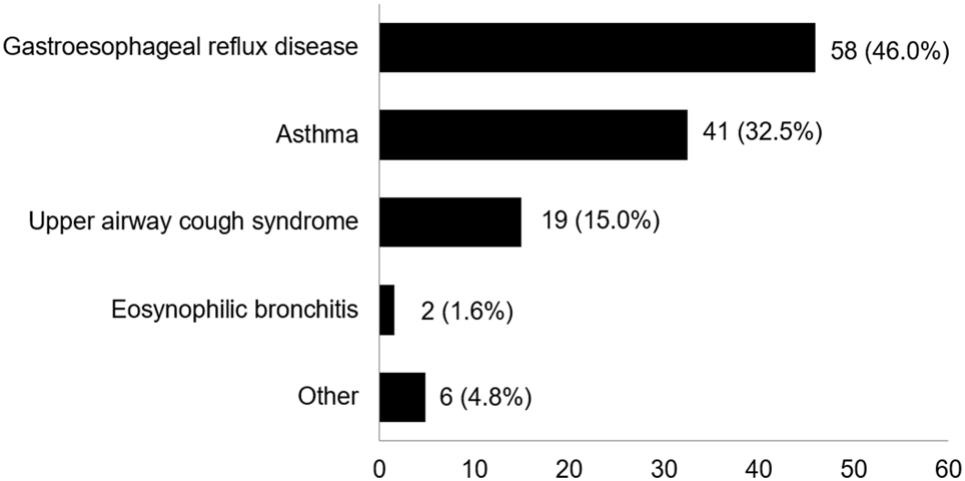
Underlying disease described as the cause of chronic cough in patients with refractory chronic cough (n = 126). Data expressed as number (%) of patients

Seventeen outpatient clinics across Spain participated in the study. A total of 203 patients were identified for participation, seven of whom were not enrolled either for failing to sign informed consent (n = 5) or current smoking (n = 2). Information was collected for 196 patients. Patients had been recruited in pulmonology (n = 166) or allergy (n = 30) departments. The population was 77.6% female, mean age was 58.5 (SD 13.3) years, and most patients (95.4%) were Caucasian. The diagnosis was RCC in 126 patients (64.3%) and UCC in 70 patients (35.7%). There were no significant differences between RCC and UCC groups with respect to age, sex distribution, race, smoking status, participation in regular exercise, body mass index distribution, or occupational status (Table 1; Supplementary Table 1). The frequency of comorbidities (unrelated to chronic cough) did not differ between RCC and UCC groups (Supplementary Table 1). GERD and asthma were the most frequent underlying diseases in patients with RCC (Fig. 1).

**Table 1:** Demographic characteristics of the study population

Characteristic		All patients (*N* = 196)	RCC (*N* = 126)	UCC (*N* = 70)	p-value (RCC *vs.* UCC)
Age, years	Mean (SD)	58.5 (13.3)	59.3 (13.0)	57.1 (13.8)	0.263
Sex	Men, n (%)	44 (22.4)	29 (23.0)	15 (21.4)	0.799
	Women, n (%)	152 (77.6)	97 (77.0)	55 (78.6)	
Body mass index, kg/m^2^	Mean (SD)	27.2 (5.0)	27.2 (4.9)	27.3 (5.1)	0.944
Smoking habit	Never, n (%)	128 (65.3)	82 (65.1)	46 (65.7)	0.929
	Past smoker, n (%)	68 (34.7)	44 (34.9)	24 (34.3)	
Regular exercise	Yes, n (%)	114 (58.2)	74 (58.7)	40 (57.1)	

See additional information in Supplementary Table 1. *RCC* refractory chronic cough; *SD* standard deviation; *UCC* unexplained chronic cough

The characteristics of cough are described in Table 2. The mean duration of cough was 6.4 (range: 1–21) years. Cough was continuous (i.e., every day or most days) in 79.6% of patients and was predominantly dry in 87.2% of patients, with no notable differences between RCC and UCC groups. A cough trigger was identified in 31.6% of patients, more frequently in those with RCC than UCC (35.5% *vs.* 22.9%; p < 0.05). As recorded in clinical charts, 37.7% of patients had atopy, and 18.4% had stress urinary incontinence (9.1% men and 21.1% women, p = 0.080).

**Table 2:** Characteristics of cough

Characteristic		All patients (*N* = 196)	RCC (*N* = 126)	UCC (*N* = 70)	p-value (RCC *vs.* UCC)
Duration of cough, years	Mean (SD)	6.4 (5.0)	7.0 (5.6)	5.3 (3.5)	0.024
Cough frequency*	Continuous, n (%)	156 (79.6)	94 (74.6)	62 (88.6)	0.024
	Intermittent, but not seasonal, n (%)	34 (17.3)	27 (21.4)	7 (10.0)	
	Intermittent, seasonal, n (%)	6 (3.1)	5 (4.0)	1 (1.4)	
Characteristics of cough	Predominantly dry, n (%)	171 (87.2)	108 (85.7)	63 (90.0)	0.389
	Predominantly productive, n (%)	25 (12.8)	18 (14.3)	7 (10.0)	
Cough triggers**	Any trigger, n (%)	62 (31.6)	46 (36.5)	16 (22.9)	0.049
	Speaking or laughing, n (%)	25 (12.8)	16 (12.7)	9 (12.9)	0.975
	Cold air or change in temperature, n (%)	21 (10.7)	14 (11.1)	7 (10.0)	0.810
	Dust, pollen, or other air irritants/particles like perfumes, n (%)	22 (11.2)	19 (15.1)	3 (4.3)	0.031
	Environmental tobacco smoke, n (%)	13 (6.6)	9 (7.1)	4 (5.7)	0.774
	Agents present at work (occupational), n (%)	2 (1.0)	2 (1.6)	0 (0.0)	0.538
	Eating or a particular type of food, n (%)	17 (8.7)	11 (8.7)	6 (8.6)	0.970
	Exercise or exertion, n (%)	13 (6.6)	9 (7.1)	4 (5.7)	0.774
	Other, n (%)	14 (7.1)	10 (7.9)	4 (5.7)	0.774
Atopy***	Yes, n (%)	66 (37.7)	50 (41.0)	16 (30.2)	0.176
Stress urinary incontinence****	Yes, n (%)	36 (18.4)	21 (16.7)	15 (21.4)	0.409

*RCC* refractory chronic cough, *SD* standard deviation, *UCC* unexplained chronic cough

^*^Continuous: Patient suffers from cough every day or nearly every day. Intermittent, but not seasonal: patient suffers periods of cough and periods of remission, but the cough is not present at specific periods in the year. Intermittent, seasonal: The patient has a chronic cough at specific times or seasons

**Percentages were calculated based on cough triggers identified in patients’ clinical charts. Patients were not interrogated on these or other potential cough triggers

***Atopy, according to local standard tests (positive skin prick test or positive determination of serum-specific IgE to aeroallergens or foods)

****Stress urinary incontinence as reflected in patients’ clinical charts

During the observation period (up to 3 years before study entry), patients had visited a range of different physicians (Table 3), most frequently pulmonologists (183 patients [93.4%], mean 5.2 [SD 4.6] visits) and primary care physicians (154 patients [78.6%], mean 5.2 [4.5] visits). Also visited frequently were ENT specialists (107 patients [54.6%], mean 2.0 [1.6] visits), allergists (90 patients [45.9%], mean 2.7 [3.9] visits), and gastroenterologists (86 patients [43.9%], mean 2.8 [2.1] visits). Twenty-four patients (12.2%) visited mental health physicians, and the mean number of visits per patient was 8.0 [11.9]). Numbers of visits per patient to various specialties did not differ between RCC and UCC groups, except for significantly more visits to gastroenterologists by patients with RCC than UCC (50.0% *vs.* 32.9%; p = 0.02). Forty patients (20.4%) had visited the hospital emergency room (mean 1.5 [0.6] visits) due to cough.

**Table 3:** Physicians visits due to chronic cough in the previous three years

Specialty		All patients (*N* = 196)	RCC (*N* = 126)	UCC (*N* = 70)	p-value (UCC *vs.* RCC)
Primary care physician	Number and percentage of patients visiting, (%)	154 (78.6)	95 (75.4)	59 (84.3)	0.146
	Mean (SD) visits per patient visiting*	5.2 (4.5)	4.9 (4.7)	5.7 (4.1)	0.276
	Mean (SD) visits per patient, all patients**	4.1 (4.5)	3.8 (4.6)	4.8 (4.3)	0.097
Pulmonologist	Number and percentage of patients visiting, (%)	183 (93.4)	114 (90.5)	69 (98.6)	0.035
	Mean (SD) visits per patient visiting*	5.2 (4.6)	5.6 (4.7)	4.6 (4.4)	0.167
	Mean (SD) visits per patient, all patients**	4.9 (4.7)	5.0 (4.8)	4.5 (4.4)	0.463
Allergist	Number and percentage of patients visiting, (%)	90 (45.9)	61 (48.4)	29 (41.4)	0.347
	Mean (SD) visits per patient visiting*	2.7 (3.9)	3.1 (4.6)	1.9 (1.7)	0.176
	Mean (SD) visits per patient, all patients**	1.2 (2.9)	1.5 (3.5)	0.8 (1.2)	0.107
ENT specialist	Number and percentage of patients visiting, (%)	107 (54.6)	64 (50.8)	43 (61.4)	0.152
	Mean (SD) visits per patient visiting*	2.0 (1.6)	1.8 (1.4)	2.2 (1.8)	0.261
	Mean (SD) visits per patient, all patients**	1.1 (1.5)	0.9 (1.4)	1.3 (1.8)	0.071
Gastroenterologist	Number and percentage of patients visiting, (%)	86 (43.9)	63 (50.0)	23 (32.9)	0.020
	Mean (SD) visits per patient visiting*	2.8 (2.1)	3.0 (2.2)	2.7 (1.7)	0.151
	Mean (SD) visits per patient, all patients**	1.2 (2.0)	1.5 (2.2)	0.7 (1.4)	0.010
Mental health (psychiatrist or psychologist)	Number and percentage of patients visiting, (%)	24 (12.2)	16 (12.7)	8 (11.4)	0.795
	Mean (SD) visits per patient visiting*	8.0 (11.9)	5.9 (4.7)	12.1 (20.0)	0.232
	Mean (SD) visits per patient, all patients**	1.0 (4.8)	0.8 (3.5)	1.4 (7.4)	0.377
Physiotherapist	Number and percentage of patients visiting, (%)	8 (4.1)	5 (4.0)	3 (4.3)	1.000
	Mean (SD) visits per patient visiting*	5.0 (6.4)	4.8 (7.4)	5.3 (5.9)	0.920
	Mean (SD) visits per patient, all patients**	0.2 (1.6)	0.2 (1.6)	0.2 (1.5)	0.871
Emergency room***	Number and percentage of patients visiting, (%)	40 (20.4)	20 (15.9)	20 (28.6)	0.035
	Mean (SD) visits per patient visiting*	1.5 (0.6)	1.6 (0.6)	1.4 (0.6)	0.295
	Mean (SD) visits per patient, all patients**	0.3 (0.6)	0.3 (0.6)	0.4 (0.7)	0.146

*ENT* ear, nose, and throat, *RCC* refractory chronic cough, *SD* standard deviation, *UCC* unexplained chronic cough

^*^Mean and SD were calculated on patients who had visited the specialist listed

**The number of visits per patient in the previous three years was calculated with the overall population as the denominator, including patients who had no visits

***In Spain, patients can attend an emergency room by their own decision, without need for referral by a physician

Five patients with RCC and one patient with UCC had been hospitalized in the previous three years, five due to complications related to chronic cough and one for investigation of chronic cough.

The main diagnostic tests performed by physicians to investigate chronic cough during the observation period are summarized in Table 4. A complete list is provided in Supplementary Table 2a-d.

**Table 4:** Diagnostic tests performed due to chronic cough in the previous three years

Diagnostic test		All patients (*N* = 196)	RCC (*N* = 126)	UCC (*N* = 70)	p-value (RCC *vs.* UCC)
a. Image					
Chest x-radiography	Number of patients (%)	164 (83.7)	108 (85.7)	56 (80.0)	0.300
	Mean (SD) tests per patient performed*	2.1 (1.7)	2.1 (1.8)	2.0 (1.3)	0.612
	Mean (SD) tests per patient, all patients**	1.8 (1.7)	1.8 (1.9)	1.6 (1.4)	0.359
X-radiography of other locations	Number of patients (%)	44 (22.4)	31 (24.6)	13 (18.6)	0.332
	Mean (SD) tests per patient performed*	2.0 (1.7)	2.0 (1.9)	2.2 (1.2)	0.745
	Mean (SD) tests per patient, all patients**	0.5 (1.2)	0.5 (1.3)	0.4 (1.0)	0.629
Chest CT scan	Number of patients (%)	100 (51.0)	69 (54.8)	31 (44.3)	0.160
	Mean (SD) tests per patient performed*	1.3 (1.0)	1.4 (1.2)	1.2 (0.5)	0.312
	Mean (SD) tests per patient, all patients**	0.7 (1.0)	0.8 (1.1)	0.5 (0.7)	0.094
b. Lung function and other lung tests					
Simple spirometry	Number of patients (%)	111 (56.6)	78 (61.9)	33 (47.1)	0.046
	Mean (SD) tests per patient performed*	1.9 (1.2)	2.0 (1.4)	1.4 (0.7)	0.017
	Mean (SD) tests per patient, all patients**	1.1 (1.3)	1.3 (1.5)	0.7 (0.9)	0.002
Spirometry with bronchodilation test	Number of patients (%)	151 (77.0)	97 (77.0)	54 (77.1)	0.980
	Mean (SD) tests per patient performed*	1.4 (0.8)	1.5 (1.0)	1.2 (0.5)	0.048
	Mean (SD) tests per patient, all patients**	1.1 (1.0)	1.1 (1.1)	0.9 (0.6)	0.126
Methacholine test	Number of patients (%)	57 (29.1)	36 (28.6)	21 (30.0)	0.833
	Mean (SD) tests per patient performed*	1.1 (0.2)	1.1 (0.3)	1.0 (0.0)	0.180
	Mean (SD) tests per patient, all patients**	0.3 (0.5)	0.3 (0.5)	0.3 (0.5)	0.898
Exhaled nitric oxide test (FeNO)	Number of patients (%)	94 (48.0)	63 (50.0)	31 (44.3)	0.443
	Mean (SD) tests per patient performed*	1.6 (1.1)	1.8 (1.2)	1.3 (0.8)	0.045
	Mean (SD) tests per patient, all patients**	0.8 (1.1)	0.9 (1.2)	0.6 (0.8)	0.055
c. Other laboratory determinations					
Total IgE Determination	Number of patients (%)	119 (60.7)	87 (69.0)	32 (45.7)	0.001
	Mean (SD) tests per patient performed*	1.4 (2.2)	1.5 (2.5)	1.1 (0.2)	0.366
	Mean (SD) tests per patient, all patients**	0.8 (1.8)	1.0 (2.2)	0.5 (0.6)	0.051
Determination of specific IgE against aeroallergens	Number of patients (%)	78 (39.8)	54 (42.9)	24 (34.3)	0.240
	Mean (SD) tests per patient performed*	1.6 (2.5)	1.3 (1.1)	2.3 (4.2)	0.116
	Mean (SD) tests per patient, all patients**	0.6 (1.8)	0.6 (0.9)	0.8 (2.7)	0.395
Skin prick testing	Number of patients (%)	103 (52.6)	64 (50.8)	39 (55.7)	0.509
	Mean (SD) tests per patient performed*	1.1 (0.4)	1.1 (0.4)	1.1 (0.2)	0.234
	Mean (SD) tests per patient, all patients**	0.6 (0.6)	0.6 (0.6)	0.6 (0.6)	0.945
d. Invasive diagnosis					
Rhinoscopy	Number of patients (%)	66 (33.7)	39 (31.0)	27 (38.6)	0.279
	Mean (SD) tests per patient performed*	1.2 (0.5)	1.3 (0.6)	1.1 (0.3)	0.209
	Mean (SD) tests per patient, all patients**	0.4 (0.7)	0.4 (0.7)	0.4 (0.6)	0.746
Bronchoscopy	Number of patients (%)	29 (14.8)	19 (15.1)	10 (14.3)	0.881
	Mean (SD) tests per patient performed*	1.0 (0.0)	1.0 (0.0)	1.0 (0.0)	(*)
	Mean (SD) tests per patient, all patients**	0.1 (0.4)	0.1 (0.4)	0.1 (0.4)	0.882
Laryngoscopy	Number of patients (%)	41 (20.9)	26 (20.6)	15 (21.4)	0.896
	Mean (SD) tests per patient performed*	1.2 (0.8)	1.3 (1.1)	1.0 (0.0)	0.266
	Mean (SD) tests per patient, all patients**	0.3 (0.6)	0.3 (0.7)	0.2 (0.4)	0.549
Upper gastrointestinal endoscopy	Number of patients (%)	54 (27.6)	41 (32.5)	13 (18.6)	0.036
	Mean (SD) tests per patient performed*	1.1 (0.4)	1.1 (4.0)	1.1 (0.3)	0.708
	Mean (SD) tests per patient, all patients**	0.3 (0.5)	0.4 (0.6)	0.2 (0.4)	0.038
Esophageal manometry/pH monitoring	Number of patients (%)	46 (23.5)	33 (26.2)	13 (18.6)	0.228
	Mean (SD) tests per patient performed*	1.1 (0.5)	1.2 (0.6)	1.0 (0.0)	0.290
	Mean (SD) tests per patient, all patients**	0.3 (0.5)	0.3 (0.6)	0.2 (0.4)	0.083

The table shows diagnostic tests performed on more than 25% of patients and invasive tests. See Supplementary Table 2 for information about additional tests, including other image and lung function tests and other laboratory and microbiological determinations. *Mean and SD were calculated on patients who had received the diagnostic test listed. **Mean number of tests per patient in the previous three years calculated with the overall population as the denominator, including patients who had not received such diagnostic test. (*) All patients underwent one bronchoscopy; the p-value cannot be calculated. *CT* computed tomography; *RCC* refractory chronic cough; *SD* standard deviation; *UCC* unexplained chronic cough

The 3-year chart review indicated that the most frequent diagnostic imaging tests were chest x-radiography in 164 patients (83.7%), a mean of 2.1 (1.7) times per patient, and chest computed tomography (CT) in 100 patients (51.0%), a mean of 1.3 (1.0) times per patient. Other imaging tests were performed less frequently (Supplementary Table 2a). There were no differences in the frequency of use of diagnostic imaging tests between patients with RCC or UCC.

The most common diagnostic lung function study was spirometry with bronchodilator test, performed in 151 patients (77.0%), a mean of 1.9 (1.2) times per patient, with no difference between the RCC and UCC groups. Simple spirometry was performed in 111 patients (56.6%), more often in those with RCC versus UCC (61.9% *vs.* 47.1%; p = 0.046). The exhaled nitric oxide (FeNO) test was used in 48.0% of patients, the methacholine test in 29.1% of patients, and the Diffusing Capacity of Lung for Carbon Monoxide (DLCO) test in 15.8% of patients, with no differences between the RCC and UCC groups. All other lung function tests were performed infrequently (Supplementary Table 2b).

Skin prick testing was performed in 103 patients (52.6%). The most frequent laboratory determinations were serum total IgE concentration (119 patients [60.7%]) and specific IgE against aeroallergens (78 patients [39.8%]). More patients with RCC than UCC (69.0% *vs.* 45.7%; p = 0.001) underwent serum total IgE concentration measurement. Tests/cultures to detect infectious diseases, Ziehl–Neelsen stained microscopy, Mantoux tests, and nasal cytology were performed infrequently, with no differences between the RCC and UCC groups (Supplementary Table 2c).

There were no differences in the proportion of patients with RCC or UCC who, during the three years before study inclusion, had undergone diagnostic rhinoscopy (overall percentage: 33.7%), bronchoscopy (14.8%), laryngoscopy (20.9%), or esophageal manometry (23.5%). Diagnostic upper digestive endoscopy was performed in a significantly greater proportion of patients with RCC than UCC (32.5% *vs.* 18.6%, p = 0.036) (Table 4; Supplementary Table 2d).

During the 3-year chart review period, the main treatments used to manage chronic cough were medications for respiratory diseases (antihistamines, inhaled corticosteroids, oral corticosteroids, inhaled bronchodilators) and proton pump inhibitors, prescribed to 87.8% and 79.5% of patients, respectively, with no differences between the RCC and UCC groups (Table 5). For most therapeutic drug classes prescribed to patients with RCC, the primary reason was ‘empirical treatment of cough’, although respiratory disease medications and proton pump inhibitors were also prescribed ‘to treat underlying disease’ or for both reasons (Supplementary Table 3). Most patients who were prescribed respiratory disease medications or proton pump inhibitors (84.6% and 84.0%, respectively) had a cumulative treatment duration of > 8 weeks (Table 5; Supplementary Table 4). Opioid-derivate cough suppressants were prescribed to 83 patients (42.3%), of whom 22 received > 8 weeks of treatment. Use of anticonvulsants or other nervous system drugs (gabapentin, pregabalin) and muscle relaxants (baclofen) was infrequent (14.3% and 6.6%, respectively) although about half of patients prescribed these medications (51.9% and 53.8%, respectively) had a cumulative treatment duration of > 8 weeks.

**Table 5:** Treatments prescribed for chronic cough in the previous three years

Treatment		All patients (*N* = 196)	RCC (*N* = 126)	UCC (*N* = 70)	p-value (RCC *vs.* UCC)*
Opioid-derivate cough suppressant drugs: codeine, dextromethorphan, dimemorfan, noscapine	Number of patients (%) who were prescribed	83 (42.3)	54 (42.9)	29 (41.4)	0.846
	Number of patients (%) who received > 8 weeks of treatment*	22 (27.2)	14 (25.9)	8 (29.6)	
Other cough suppressant drugs: levodropropizine, cloperastine	Number of patients (%) who were prescribed	24 (12.2)	10 (7.9)	14 (20.0)	0.014
	Number of patients (%) who received > 8 weeks of treatment*	10 (16.1)	9 (22.5)	1 (4.5)	
Expectorants: guaifenesin or others	Number of patients (%) who were prescribed	12 (6.1)	8 (6.3)	4 (5.7)	1.000
	Number of patients (%) who received > 8 weeks of treatment*	2 (16.7)	1 (12.5)	1 (25.0)	
Mucolytics: acetylcysteine, ambroxol, bromhexine, carbocisteine, or others	Number of patients (%) who were prescribed	65 (33.2)	42 (33.3)	23 (32.9)	0.946
	Number of patients (%) who received > 8 weeks of treatment*	10 (16.1)	9 (22.5)	1 (4.5)	
Anticonvulsants or other nervous system drugs: gabapentin, pregabalin	Number of patients (%) who were prescribed	28 (14.3)	15 (11.9)	13 (18.6)	0.201
	Number of patients (%) who received > 8 weeks of treatment*	14 (51.9)	8 (53.3)	6 (50.0)	
Muscle relaxants (baclofen)	Number of patients (%) who were prescribed	13 (6.6)	7 (5.6)	6 (8.6)	0.416
	Number of patients (%) who received > 8 weeks of treatment*	7 (53.8)	2 (28.6)	5 (83.3)	
Proton pump inhibitors	Number of patients (%) who were prescribed	156 (79.6)	104 (82.5)	52 (74.3)	0.169
	Number of patients (%) who received > 8 weeks of treatment*	126 (84.0)	90 (88.2)	36 (75.0)	
Therapies used to treat other respiratory diseases: antihistamines, inhaled corticosteroids, oral corticosteroids, inhaled bronchodilators (beta-agonists, anticholinergics)	Number of patients (%) who were prescribed	172 (87.8)	110 (87.3)	62 (88.6)	0.795
	Number of patients (%) who received > 8 weeks of treatment*	143 (84.6)	95 (87.2)	48 (80.0)	

Therapeutic families were classified according to the classification of the Ministry of Health of Spain. Reasons for prescribing therapies in patients with RCC are provided in Supplementary Table 3

*RCC* refractory chronic cough; *UCC* unexplained chronic cough

*See Supplementary Table 4 for more details about the cumulative duration of therapy and p-values

**Fig. 2:**
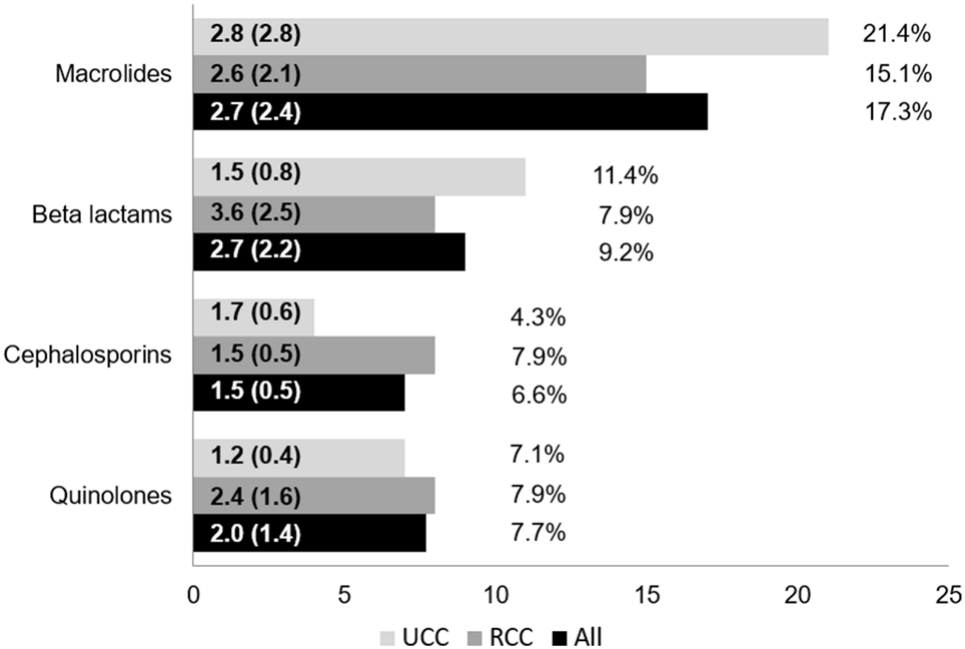
Percentage of patients receiving antibiotic therapies for empirical treatment of chronic cough and number of antibiotic cycles. Numbers in the bars represent the mean (standard deviation) number of cycles received per patient who received a specific antibiotic from each class. Percentages reflect the percentage of patients who had received such an antibiotic at least once to treat chronic cough without evidence of underlying infection. *RCC* refractory chronic cough, *UCC* unexplained chronic cough

Over the previous three years, 56 patients (28.6%) had been prescribed antibiotics for empirical treatment of chronic cough (i.e., without evidence of underlying infectious disease). The proportion was numerically but not significantly higher in the UCC than RCC group (34.5% *vs.* 25.4%; p = 0.187). Macrolides were the antibiotic family prescribed most often. Among 18 patients (9.2%) who were prescribed non-cephalosporin beta-lactams (e.g., amoxicillin and others), the number of courses per patient was higher in the RCC versus UCC group (3.6 *vs.* 1.5 courses; p = 0.040). This pattern was not observed with other antibiotic classes (Fig. 2).

## Discussion

This retrospective study aimed to evaluate the characteristics and management approach to RCC and UCC in Spanish outpatient clinics to better understand the consequences and burden of these conditions to patients and the healthcare system. The study provides quantitative data about health resource utilization associated with the diagnosis and management of RCC and UCC, as recorded in patients’ medical records. Data reported by Spanish patients who participated in the cross-sectional European National Health and Wellness Survey applied to chronic cough in general [4], whereas our study is specific to the difficult-to-manage subsets of patients with RCC or UCC. It is important to emphasize that the results derive from patients who were being followed for their RCC or UCC in outpatient hospital clinics. Population-based studies of health-seeking behavior due to cough found that a substantial proportion (60%) of individuals with chronic cough do not seek medical treatment for their condition [15–17]. In Spain, there are no cough clinics per se that can contribute a large number of patients to investigation. Thus, a relatively large number of centers participated in the present study. The inclusion period had to be extended to 19 months due to restrictions imposed by the COVID 19 pandemic.

Our cohort was similar with respect to gender distribution and mean age as other chronic cough populations participating in noninterventional studies [18, 19] or randomized controlled trials [20, 21]. The main difference between RCC and UCC groups was that, by definition, RCC patients had a known cough-associated condition (e.g., GERD, asthma), which was ultimately reflected by between-group variation in both the type and number of physician visits and diagnostic tests.

Our study confirmed that RCC and UCC are responsible for substantial health resource consumption in Spain. More than 90% of patients had visited a pulmonologist, and approximately 80% of patients had visited a primary care physician, for a cough-related reason an average of five times per specialist during the previous three years. One in five patients (more with UCC than RCC) had visited an emergency department due to cough. Although only 12% of patients had sought mental health services (psychiatrist, psychologist), the mean (SD) number of visits per patient (8.0 [11.9]) over the 3-year observation period was the highest among specialties.

An expert panel has advocated for clinicians treating chronic cough to practice ‘intervention fidelity’, which is described as adhering closely to best practice diagnostic and treatment guidelines [13]. The high observed usage of chest X-rays and spirometry as diagnostic tests aligns with guideline recommendations for a diagnostic work-up of chronic cough [10, 11]. Likewise, frequent use of chest CT scans, IgE determination, and skin prick testing suggested that physicians were attempting to identify cough-associated conditions or treatable traits as per European guideline recommendations [10]. More frequent use of total IgE determination and upper gastrointestinal endoscopy in RCC than UCC patients was likely due to the presence of asthma and/or GERD in most of the RCC group.

Medications prescribed to RCC and UCC patients reflected the treatment repertoire for chronic cough at the time of the study and aligned broadly with management recommendations [10, 11]. Consistent with GERD and asthma as common cough-associated conditions [10, 11], the most frequently prescribed therapeutic classes to treat an underlying disease or for empirical reasons (or both) were proton pump inhibitors and respiratory medications. A cumulative treatment duration of > 8 weeks in most patients who were prescribed these medications suggests a reasonable trial of therapy. In contrast, the prescribing of opioid-derivate cough suppressants to 42% of patients, and a cumulative treatment duration of > 8 weeks in 27% of this group, may be of concern given the risks and adverse events associated with extended use of opioids, although this is unclear in the absence of any detail about the quantity and frequency of administration. The use of nonspecific therapies (e.g., anticonvulsants, muscle relaxants), mainly for empirical purposes, was low, which is not unexpected given that their effectiveness in alleviating chronic cough is unclear and tolerability can be poor [11]. Interestingly, despite the wide range of medications prescribed to treat chronic cough, patients still presented cough at study enrolment (as per inclusion criteria), which was described as continuous (i.e., every day or nearly every day) in 80% of subjects, highlighting the need for more effective therapies to treat RCC or UCC.

Chronic cough appears to be a driver for inappropriate use of antibiotics. Antibiotics were used empirically in more than one-quarter of patients, despite being indicated only to treat an underlying infectious disease such as sinusitis or chronic bronchitis refractory to other therapy [10, 11]. As such, there is a continued need to improve antibiotic stewardship in the outpatient setting to avoid unnecessary or inappropriate use that can favor the development of antimicrobial resistance [22, 23].

Study limitations include those inherent to observational studies, such as selection bias, lack of a control group, and incomplete or missing data. Patients who seek and subsequently maintain medical treatment for chronic cough represent a minority of the chronic cough population. There were no established criteria other than physicians’ clinical judgment (based on patients’ history, diagnostic test results and therapies used) to classify patients as RCC or UCC in this study. Although unlikely, we cannot rule out some misclassification. Selection bias was mitigated to some extent by enrolling consecutive patients, although patients with serious conditions are more frequent attendees at outpatient clinics and thus have a greater chance of being enrolled. There may be inaccuracies in the data as the primary source was patients’ medical records without prospective confirmation. Visits to primary care physicians in particular may be underestimated as several outpatient hospital clinics had no easy access to this information. Moreover, because we studied only the three years prior to enrollment, the frequency of specialist visits, diagnostic tests, and prescribed treatments reflects this time period, not the entire patient history, and may also be underestimated. For the same reason, the study reflects only 83.7% of patients having undergone chest radiography; the remainder are assumed to have had this essential test performed before the study observation period. The mean 6.4 (5.0) year duration of chronic cough in the cohort suggests that, in some patients, the bulk of the diagnostic work-up and associated health resource utilization had taken place before our three-year chart review period.

In recent years, chronic cough has become increasingly recognized as a condition of neural dysregulation [7]. Most patients with chronic cough have cough reflex hypersensitivity, which is characterized by a heightened neural responsivity to various stimuli affecting the airway and lungs [11]. The shift in perception from cough as a consequence of underlying disease to cough as a distinct clinical entity has paved the way for development of novel antitussives that act on specific cough pathways [24]. Pathophysiological underlying mechanisms such as ATP release, which may stimulate vagal C-fiber afferent sensory neurons upon binding to purinergic receptors, including the P2X3 receptor, have been identified as contributors to chronic cough [25]. This has led to the development of P2X3 receptor antagonists with potential efficacy for treating RCC and UCC and alleviating patients from the burden of excessive cough [26].

## Conclusion

This retrospective study, which aimed to profile patients with RCC and UCC and determine their health resource utilization, confirms that these conditions pose a substantial burden to healthcare systems in Spain. Despite broad alignment between European guideline recommendations for treating chronic cough and the management approaches undertaken by Spanish physicians, the findings highlight the shortcomings of available treatments, which mainly do not address the underlying pathology of cough. Recognizing cough reflex hypersensitivity as a clinical feature may help identify patients with potential to benefit from new therapies that target specific receptors in the cough pathway. These newer therapies offer opportunities for more effective management of RCC and UCC to the benefit of patients and healthcare systems.

## Supplementary Information

Below is the link to the electronic supplementary material.

Supplementary file1 (DOCX 48 KB)
