# Supplementary material for: Characteristics and Management of Patients with Refractory or Unexplained Chronic Cough in Outpatient Hospital Clinics in Spain: A Retrospective Multicenter Study
Source: Lung. 2023 May 9;201(3):275–86. doi: 10.1007/s00408-023-00620-y (PMC10169201; doi:10.1007/s00408-023-00620-y)
Supplement: Supplementary file 1 — Supplementary file1 (DOCX 48 KB) [file 408_2023_620_MOESM1_ESM.docx]

**Supplementary Table 1** Demographic characteristics and comorbidities

| **Characteristic** | | **All patients**  **(*N* = 196)** | **RCC**  **(*N* = 126)** | **UCC**  **(*N* = 70)** | **p-value**  **(RCC *vs* UCC)** |
| --- | --- | --- | --- | --- | --- |
| **Occupation** | Student, n (%) | 4 (2.0) | 1 (0.8) | 3 (4.3) | 0.087 |
|  | Active worker, n (%) | 85 (43.4) | 56 (44.4) | 29 (41.4) |  |
|  | On sick leave, n (%) | 11 (5.6) | 6 (4.8) | 5 (7.1) |  |
|  | Unemployed, n (%) | 11 (5.6) | 4 (3.2) | 7 (10.0) |  |
|  | Housekeeping, n (%) | 21 (10.7) | 17 (13.5) | 4 (5.7) |  |
|  | Retired, n (%) | 64 (32.7) | 42 (33.3) | 22 (31.4) |  |
| **Body mass index classification** | Underweight, n (%) | 2 (1.0) | 2 (1.6) | 0 (0.0) | 0.279 |
|  | Normal weight, n (%) | 68 (35.4) | 41 (33.1) | 27 (39.7) |  |
|  | Overweight, n (%) | 67 (34.9) | 48 (38.7) | 19 (27.9) |  |
|  | Obese, n (%) | 54 (28.1) | 33 (26.6) | 21 (30.9) |  |
|  | Morbidly obese, n (%) | 1 (0.5) | 0 (0.0) | 1 (1.5) |  |
| **Arterial hypertension** | n (%) | 56 (28.6) | 36 (28.6) | 20 (28.6) | 1.000 |
| **Diabetes mellitus** | n (%) | 15 (7.7) | 12 (9.5) | 3 (4.3) | 0.186 |
| **Cardiovascular disease** | n (%) | 14 (7.1) | 12 (9.5) | 2 (2.9) | 0.082 |
| **Cerebrovascular disease** | n (%) | 5 (2.6) | 3 (2.4) | 2 (2.9) | 1.000 |
| **Chronic hepatic disease** | n (%) | 3 (1.5) | 3 (2.4) | 0 (0.0) | 0.554 |
| **Rheumatic diseases** | n (%) | 13 (6.6) | 9 (7.1) | 4 (5.7) | 0.774 |
| **Cancer (excluding lung or upper respiratory airway)** | n (%) | 9 (4.6) | 5 (4.0) | 4 (5.7) | 0.724 |

There were no cases of degenerative neurological disease. *RCC* refractory chronic cough; *UCC* unexplained chronic cough.

**Supplementary** **Table 2a-d** Diagnostic tests performed due to chronic cough in the previous three years (complete table)

| **a. Image** | | **All patients**  **(*N* = 196)** | **RCC**  **(*N* = 126)** | **UCC**  **(*N* = 70)** | **p-value**  **(RCC *vs* UCC)** |
| --- | --- | --- | --- | --- | --- |
| **Chest x-radiography** | Number of patients (%) | 164 (83.7) | 108 (85.7) | 56 (80.0) | 0.300 |
|  | Mean (SD) tests per patient performed* | 2.1 (1.7) | 2.1 (1.8) | 2.0 (1.3) | 0.612 |
|  | Mean (SD) tests per patient, all patients** | 1.8 (1.7) | 1.8 (1.9) | 1.6 (1.4) | 0.359 |
| **X-radiography of other location** | Number of patients (%) | 44 (22.4) | 31 (24.6) | 13 (18.6) | 0.332 |
|  | Mean (SD) tests per patient performed* | 2.0 (1.7) | 2.0 (1.9) | 2.2 (1.2) | 0.745 |
|  | Mean (SD) tests per patient, all patients** | 0.5 (1.2) | 0.5 (1.3) | 0.4 (1.0) | 0.629 |
| **Chest CT scan** | Number of patients (%) | 100 (51.0) | 69 (54.8) | 31 (44.3) | 0.160 |
|  | Mean (SD) tests per patient performed* | 1.3 (1.0) | 1.4 (1.2) | 1.2 (0.5) | 0.312 |
|  | Mean (SD) tests per patient, all patients** | 0.7 (1.0) | 0.8 (1.1) | 0.5 (0.7) | 0.094 |
| **Chest magnetic resonance** | Number of patients (%) | 2.0 (1.0) | 2.0 (1.6) | 0.0 (0.0) | 0.538 |
|  | Mean (SD) tests per patient performed* | 2.0 (1.4) | 2.0 (1.4) | - | (*) |
|  | Mean (SD) tests per patient, all patients** | 0.0 (0.2) | 0.0 (0.3) | 0.0 (0.0) | 0.346 |
| **CT scan of other location** | Number of patients (%) | 21 (10.7) | 17 (13.5) | 4 (5.7) | 0.146 |
|  | Mean (SD) tests per patient performed* | 1.5 (1.2) | 1.5 (1.2) | 1.3 (0.5) | 0.666 |
|  | Mean (SD) tests per patient, all patients** | 0.2 (0.6) | 0.2 (0.7) | 0.0 (0.3) | 0.120 |
| **Magnetic resonance of other location** | Number of patients (%) | 7 (3.6) | 5 (4.0) | 2.0 (2.9) | 1.000 |
|  | Mean (SD) tests per patient performed* | 1.7 (0.8) | 1.6 (0.5) | 2.0 (1.4) | 0.576 |
|  | Mean (SD) tests per patient, all patients** | 0.1 (0.3) | 0.1 (0.3) | 0.0 (0.4) | 0.902 |
| **b. Lung function and other lung tests** | | **All patients**  **(*N* = 196)** | **RCC**  **(*N* = 126)** | **UCC**  **(*N* = 70)** | **p-value**  **(RCC *vs* UCC)** |
| **Simple spirometry** | Number of patients (%) | 111 (56.6) | 78 (61.9) | 33 (47.1) | 0.046 |
|  | Mean (SD) tests per patient performed* | 1.9 (1.2) | 2.0 (1.4) | 1.4 (0.7) | 0.017 |
|  | Mean (SD) tests per patient, all patients** | 1.1 (1.3) | 1.3 (1.5) | 0.7 (0.9) | 0.002 |
| **Spirometry with bronchodilation test** | Number of patients (%) | 151 (77.0) | 97 (77.0) | 54 (77.1) | 0.980 |
|  | Mean (SD) tests per patient performed* | 1.4 (0.8) | 1.5 (1.0) | 1.2 (0.5) | 0.048 |
|  | Mean (SD) tests per patient, all patients** | 1.1 (1.0) | 1.1 (1.1) | 0.9 (0.6) | 0.126 |
| **Methacholine test** | Number of patients (%) | 57 (29.1) | 36 (28.6) | 21 (30.0) | 0.833 |
|  | Mean (SD) tests per patient performed* | 1.1 (0.2) | 1.1 (0.3) | 1.0 (0.0) | 0.180 |
|  | Mean (SD) tests per patient, all patients** | 0.3 (0.5) | 0.3 (0.5) | 0.3 (0.5) | 0.898 |
| **Mannitol test** | Number of patients (%) | 6 (3.1) | 2 (1.6) | 4 (5.7) | 0.189 |
|  | Mean (SD) tests per patient performed* | 1.2 (0.4) | 1.5 (0.7) | 1.0 (0.0) | 0.178 |
|  | Mean (SD) tests per patient, all patients** | 0.0 (0.2) | 0.0 (0.2) | 0.1 (0.2) | 0.292 |
| **Exhaled nitric oxide test (FeNO)** | Number of patients (%) | 94 (48.0) | 63 (50.0) | 31 (44.3) | 0.443 |
|  | Mean (SD) tests per patient performed* | 1.6 (1.1) | 1.8 (1.2) | 1.3 (0.8) | 0.045 |
|  | Mean (SD) tests per patient, all patients** | 0.8 (1.1) | 0.9 (1.2) | 0.6 (0.8) | 0.055 |
| **Capsaicin test** | Number of patients (%) | 2 (1.0) | 2 (1.6) | 0 (0.0) | 0.538 |
|  | Mean (SD) tests per patient performed* | 1.5 (0.7) | 1.5 (0.7) | - | (*) |
|  | Mean (SD) tests per patient, all patients** | 0.0 (0.2) | 0.0 (0.2) | 0.0 (0.0) | 0.318 |
| **Specific inhalation challenge test** | Number of patients (%) | 12 (6.1) | 9 (7.1) | 3 (4.3) | 0.543 |
|  | Mean (SD) tests per patient performed* | 1.0 (0.0) | 1.0 (0.0) | 1.0 (0.0) | (*) |
|  | Mean (SD) tests per patient, all patients** | 0.1 (0.2) | 0.1 (0.3) | 0.0 (0.2) | 0.427 |
| **CO diffusion capacity test (DLCO)** | Number of patients (%) | 31 (15.8) | 19 (15.1) | 12 (17.1) | 0.704 |
|  | Mean (SD) tests per patient performed* | 1.1 (0.3) | 1.0 (0.0) | 1.3 (0.4) | 0.021 |
|  | Mean (SD) tests per patient, all patients** | 0.1 (0.2) | 0.2 (0.4) | 0.2 (0.5) | 0.310 |
| **Plethysmography** | Number of patients (%) | 18 (9.2) | 13 (10.3) | 5 (7.1) | 0.461 |
|  | Mean (SD) tests per patient performed* | 1.0 (0.0) | 1.0 (0.0) | 1.0 (0.0) | (*) |
|  | Mean (SD) tests per patient, all patients** | 0.1 (0.3) | 0.1 (0.3) | 0.1 (0.3) | 0.463 |
| **Sputum induction** | Number of patients (%) | 8 (4.1) | 7 (5.6) | 1 (1.4) | 0.263 |
|  | Mean (SD) tests per patient performed* | 1.5 (0.8) | 1.4 (0.8) | 2.0 (-) | 0.522 |
|  | Mean (SD) tests per patient, all patients** | 0.1 (0.3) | 0.1 (/0.4) | 0.0 (0.2) | 0.303 |
| **c. Other lab determinations** | | **All patients**  **(*N* = 196)** | **RCC**  **(*N* = 126)** | **UCC**  **(*N* = 70)** | **p-value**  **(RCC *vs* UCC)** |
| **Total IgE determination** | Number of patients (%) | 119 (60.7) | 87 (69.0) | 32 (45.7) | 0.001 |
|  | Mean (SD) tests per patient performed* | 1.4 (2.2) | 1.5 (2.5) | 1.1 (0.2) | 0.366 |
|  | Mean (SD) tests per patient, all patients** | 0.8 (1.8) | 1.0 (2.2) | 0.5 (0.6) | 0.051 |
| **Determination of specific IgE against aeroallergens** | Number of patients (%) | 78 (39.8) | 54 (42.9) | 24 (34.3) | 0.240 |
|  | Mean (SD) tests per patient performed* | 1.6 (2.5) | 1.3 (1.1) | 2.3 (4.2) | 0.116 |
|  | Mean (SD) tests per patient, all patients ** | 0.6 (1.8) | 0.6 (0.9) | 0.8 (2.7) | 0.395 |
| **Infectious diseases detecting tests or cultures (*Bordetella*, *Chlamydia*, *Mycoplasma* or any other)** | Number of patients (%) | 28 (14.3) | 20 (15.9) | 8 (11.4) | 0.394 |
|  | Mean (SD) tests per patient performed* | 2.1 (2.0) | 2.4 (2.3) | 1.6 (0.7) | 0.404 |
|  | Mean (SD) tests per patient, all patients** | 0.3 (1.1) | 0.4 (1.3) | 0.2 (0.6) | 0.240 |
| **Ziehl–Neelsen stained microscopy** | Number of patients (%) | 22 (11.2) | 17 (13.5) | 5 (7.1) | 0.177 |
|  | Mean (SD) tests per patient performed* | 1.6 (0.7) | 1.5 (0.7) | 1.8 (0.8) | 0.482 |
|  | Mean (SD) tests per patient, all patients** | 0.2 (0.6) | 0.2 (0.6) | 0.1 (0.5) | 0.351 |
| **Mantoux** | Number of patients (%) | 5 (2.6) | 5 (4.0) | 0 (0.0) | 0.162 |
|  | Mean (SD) tests per patient performed* | 1.0 (0.0) | 1.0 (0.0) | - | (*) |
|  | Mean (SD) tests per patient, all patients** | 0.0 (0.2) | 0.0 (0.2) | 0.0 (0.0) | 0.092 |
| **Nasal cytology** | Number of patients (%) | 1 (0.5) | 0 (0.0) | 1 (1.4) | 0.357 |
|  | Mean (SD) tests per patient performed* | 1.0 (-) | - | 1.0 (-) | (*) |
|  | Mean (SD) tests per patient, all patients** | 0.0 (0.1) | 0.0 (0.0) | 0.0 (0.1) | 0.180 |
| **Skin prick testing** | Number of patients (%) | 103 (52.6) | 64 (50.8) | 39 (55.7) | 0.509 |
|  | Mean (SD) tests per patient performed* | 1.1 (0.4) | 1.1 (0.4) | 1.1 (0.2) | 0.234 |
|  | Mean (SD) tests per patient, all patients** | 0.6 (0.6) | 0.6 (0.6) | 0.6 (0.6) | 0.945 |
| **d. Invasive diagnosis** | | **All patients**  **(*N* = 196)** | **RCC**  **(*N* = 126)** | **UCC**  **(*N* = 70)** | **p-value**  **(RCC *vs* UCC)** |
| **Rhinoscopy** | Number of patients (%) | 66 (33.7) | 39 (31.0) | 27 (38.6) | 0.279 |
|  | Mean (SD) tests per patient performed* | 1.2 (0.5) | 1.3 (0.6) | 1.1 (0.3) | 0.209 |
|  | Mean (SD) tests per patient, all patients** | 0.4 (0.7) | 0.4 (0.7) | 0.4 (0.6) | 0.746 |
| **Bronchoscopy** | Number of patients (%) | 29 (14.8) | 19 (15.1) | 10 (14.3) | 0.881 |
|  | Mean (SD) tests per patient performed* | 1.0 (0.0) | 1.0 (0.0) | 1.0 (0.0) | (*) |
|  | Mean (SD) tests per patient, all patients** | 0.1 (0.4) | 0.1 (0.4) | 0.1 (0.4) | 0.882 |
| **Laryngoscopy** | Number of patients (%) | 41 (20.9) | 26 (20.6) | 15 (21.4) | 0.896 |
|  | Mean (SD) tests per patient performed* | 1.2 (0.8) | 1.3 (1.1) | 1.0 (0.0) | 0.266 |
|  | Mean (SD) tests per patient, all patients** | 0.3 (0.6) | 0.3 (0.7) | 0.2 (0.4) | 0.549 |
| **Upper gastrointestinal endoscopy** | Number of patients (%) | 54 (27.6) | 41 (32.5) | 13 (18.6) | 0.036 |
|  | Mean (SD) tests per patient performed* | 1.1 (0.4) | 1.1 (4.0) | 1.1 (0.3) | 0.708 |
|  | Mean (SD) tests per patient, all patients** | 0.3 (0.5) | 0.4 (0.6) | 0.2 (0.4) | 0.038 |
| **Esophageal manometry / pH monitoring** | Number of patients (%) | 46 (23.5) | 33 (26.2) | 13 (18.6) | 0.228 |
|  | Mean (SD) tests per patient performed* | 1.1 (0.5) | 1.2 (0.6) | 1.0 (0.0) | 0.290 |
|  | Mean (SD) tests per patient, all patients** | 0.3 (0.5) | 0.3 (0.6) | 0.2 (0.4) | 0.083 |

* Mean and SD calculated on patients who had visited the specialist listed. **Mean number of tests per patient in the previous 3 years calculated with the overall population as denominator, including patients who had not received such a diagnostic test. (*) p-value cannot be calculated due to lack of variance or because in one of the groups no patient had the listed test performed. *CO* carbon monoxide; *CT* computed tomography; *RCC* refractory chronic cough; *SD* standard deviation; *UCC* unexplained chronic cough.

**Supplementary Table 3** Therapies used to treat chronic cough in the previous three years. Reason for prescription in patients with RCC*

| **Treatment** | **Reason for prescription** |  |
| --- | --- | --- |
| **Opioid-derivate cough suppressant drugs (R05DA): codeine, dextromethorphan, dimemorfan, noscapine** | Treatment of underlying disease, n (%) | 0 (0.0) |
|  | Empirical treatment of cough, n (%) | 50 (92.6) |
|  | Both, n (%) | 4 (7.4) |
| **Other cough suppressant drugs (R05DB): levodropropizine, cloperastine** | Treatment of underlying disease, n (%) | 0 (0.0) |
|  | Empirical treatment of cough, n (%) | 9 (90.0) |
|  | Both, n (%) | 1 (10.0) |
| **Expectorants (R05CA): guaifenesin or others** | Treatment of underlying disease, n (%) | 1 (12.5) |
|  | Empirical treatment of cough, n (%) | 6 (75.0) |
|  | Both, n (%) | 1 (12.5) |
| **Mucolytics (R05CB): acetylcysteine, ambroxol, bromhexine, carbocisteine or others** | Treatment of underlying disease, n (%) | 7 (16.7) |
|  | Empirical treatment of cough, n (%) | 29 (69.0) |
|  | Both, n (%) | 6 (14.3) |
| **Anticonvulsants or other nervous system drugs: gabapentin, pregabalin** | Treatment of underlying disease, n (%) | 4 (20.0) |
|  | Empirical treatment of cough, n (%) | 11 (73.3) |
|  | Both, n (%) | 1 (6.7) |
| **Muscle relaxants (baclofen)** | Treatment of underlying disease, n (%) | 0 (0.0) |
|  | Empirical treatment of cough, n (%) | 5 (71.4) |
|  | Both, n (%) | 2 (28.6) |
| **Proton pump inhibitors** | Treatment of underlying disease, n (%) | 45 (43.3) |
|  | Empirical treatment of cough, n (%) | 31 (29.8) |
|  | Both, n (%) | 28 (26.9) |
| **Therapies used to treat other respiratory diseases: antihistamines, inhaled corticosteroids, oral corticosteroids, inhaled bronchodilators (beta-agonists, anticholinergics)** | Treatment of underlying disease, n (%) | 36 (32.7) |
|  | Empirical treatment of cough, n (%) | 42 (38.2) |
|  | Both, n (%) | 32 (29.1) |

*Percentages are calculated based on the number of patients with RCC who had been prescribed each therapy and had information available about the reason for prescription. *RCC* refractory chronic cough.

**Supplementary Table 4** Therapies used to treat chronic cough in the previous three years. Cumulative duration of therapy*

| **Treatment** | **Cumulative duration of treatment** | **RCC** | **UCC** | **p-value** |
| --- | --- | --- | --- | --- |
| **Opioid-derivate cough suppressant drugs (R05DA): codeine, dextrometorfan, dimemorfan, noscapine** | Less than three weeks, n (%) | 26 (48.1) | 10 (37.0) | 0.437 |
|  | Three to eight weeks, n (%) | 14 (25.9) | 9 (33.3) |  |
|  | More than eight weeks, n (%) | 14 (25.9) | 8 (29.6) |  |
| **Other cough suppressant drugs (R05DB): levodropropizine, cloperastine** | Less than three weeks, n (%) | 3 (30.0%) | 4 (30.8) | 0.431 |
|  | Three to eight weeks, n (%) | 2 (20.0%) | 7 (53.8) |  |
|  | More than eight weeks, n (%) | 5 (50.0%) | 2 (15.4) |  |
| **Expectorants (R05CA): guaifenesin or others** | Less than three weeks, n (%) | 3 (37.5) | 3 (75.0) | 0.527 |
|  | Three to eight weeks, n (%) | 4 (50.0) | 0 (0.0) |  |
|  | More than eight weeks, n (%) | 1 (12.5) | 1 (25.0) |  |
| **Mucolytics (R05CB): acetylcysteine, ambroxol, bromhexine, carbocisteine or others** | Less than three weeks, n (%) | 19 (47.5) | 10 (45.5) | 0.431 |
|  | Three to eight weeks, n (%) | 12 (30.0) | 11 (50.0) |  |
|  | More than eight weeks, n (%) | 9 (22.5) | 1 (4.5) |  |
| **Anticonvulsants or other nervous system drugs: gabapentin, pregabalin** | Less than three weeks, n (%) | 2 (13.3) | 2 (16.7) | 0.880 |
|  | Three to eight weeks, n (%) | 5 (33.3) | 4 (33.3) |  |
|  | More than eight weeks, n (%) | 8 (53.3) | 6 (50.0) |  |
| **Muscle relaxants (baclofen)** | Less than three weeks, n (%) | 2 (28.6) | 0 (0.0) | 0.078 |
|  | Three to eight weeks, n (%) | 3 (42.9) | 1 (16.7) |  |
|  | More than eight weeks, n (%) | 2 (28.6) | 5 (83.3) |  |
| **Proton pump inhibitors** | Less than three weeks, n (%) | 2 (2.0) | 2 (4.2) | 0.048 |
|  | Three to eight weeks, n (%) | 10 (9.8) | 10 (20.8) |  |
|  | More than eight weeks, n (%) | 90 (88.2) | 36 (75.0) |  |
| **Therapies used for treatment of other respiratory diseases: antihistamines, inhaled corticosteroids, oral corticosteroids, inhaled bronchodilators (beta-agonists, anticholinergic)** | Less than three weeks, n (%) | 3 (2.8) | 2 (3.3) | 0.249 |
|  | Three to eight weeks, n (%) | 11 (10.1) | 10 (16.7) |  |
|  | More than eight weeks, n (%) | 95 (87.2) | 48 (80.0) |  |

*Percentages are calculated based on the number of patients who had been prescribed each therapy and had information available about the cumulative duration of therapy. *ND* no data; *RCC* refractory chronic cough; *UCC* unexplained chronic cough.
